# Epistemic communities and experts in health policy-making

**DOI:** 10.1093/eurpub/cky156

**Published:** 2018-11-01

**Authors:** Olga Löblová

**Affiliations:** Department of Sociology, University of Cambridge, Cambridge, UK

## Abstract

The role of evidence and expertise in policy-making has been of interest to public health professionals and political scientists alike. The public health community often sees its efforts as part of a linear knowledge transfer process and tends to blame itself for inadequate communication or translation of its arguments to policy-makers’ language when its efforts fail. Political science, especially theories of the policy process, offer alternative perspectives to explain the success or failure of experts’ preferred policy goals. This paper focuses on the concept of epistemic communities (groups of experts with a common policy goal derived from their shared knowledge) in policy-making, drawing on examples from the field of health technology assessment in Europe. By combining the parsimony and the central focus on experts of the linear knowledge transfer model with the recognition of complexity of political science, the epistemic communities concept provides a useful structure for the public health community to analyze its efforts to influence policy.

## Introduction

Knowledge, evidence and expert advice are crucial for rational policy-making in the modern state. In health policy, they have had an especially prominent role following the rise of evidence-based medicine and its extension to evidence-based, or evidence-informed, policy.^1^^,^^2^ The role of experts in policy-making has long been of interest to political scientists, as well as to the experts themselves: how is it possible that expert advice sometimes seems to determine policy, only to be blatantly disregarded on other occasions?

Experts often see themselves as heroes in an epic: they must swim seven seas (‘bridge gaps between science and policy’), climb seven mountains (‘overcome barriers to adoption of research’) and translate their wisdom into a foreign tongue for the king to understand (‘engage in knowledge translation’). When they fail, their default response is to try harder: set up more knowledge transfer initiatives, employ more science communication professionals. In contrast, political science offers more nuanced, and perhaps more comforting, ways of thinking about science and policy. This paper focuses on a particular conceptualization of experts as *epistemic communities*: groups of experts with a common policy goal derived from their shared knowledge.^3^ The epistemic communities concept has the advantage of being relatively similar to the ‘hero’s journey’ linear model of science–policy interaction often used by the public health community. At the same time, it allows for incorporating the complexity and idiosyncrasy of the policy process emphasized by political science perspectives.

To illustrate its theoretical points, the paper draws on examples from the spread of health technology assessment (HTA) in European countries and at the European Union (EU) level.^4^^,^^5^ As a multi-disciplinary evaluation of available evidence on the medical, economic, ethical, legal, social and other aspects of health interventions, HTA has proven to be a very popular policy tool for informing coverage decisions in the past 20 years. During this time, an EU-level HTA has become a staple of Brussels health policy debates, and specialized national or regional HTA bodies have been set up in many EU member states. Domestic, as well as European, epistemic communities have played a major role in their establishment.

## Experts and policy: from linear models to complexity

The linear view of experts’ influence on policy-making simplifies the relationship to a ‘one-way process where researchers produce new knowledge, which then gets disseminated to end users and finally is incorporated in policy and practice’.^6^ In other words, experts and policy-makers are two distinct communities, separated by barriers and gaps that need to be bridged in order to achieve evidence-based policy.^7^ Policy itself is seen as a black box or a nuisance—‘an abstract construct best left to politicians, or as a distal determinant of health that can be changed following Cartesian heuristics’^8^—and if it ends up ignoring evidence, it is because the two communities do not speak the same language and scientists have not managed to convey their message in simple enough terms. As a result, scientists need to develop a range of strategies to improve ‘knowledge transfer’, (or ‘knowledge translation’) from their own ranks to policy-makers, increase ‘knowledge utilization’, and create specialized actors, e.g. ‘knowledge brokers’.^9^ The public health community is not immune to this linear-rational view of its efforts; in fact, it is more preoccupied than experts from other areas with barriers and facilitators to the use of evidence in policy.^10^

In the linear model, scientists retain an internal locus of control. As documented by the numerous practical tips on improving communication with policy-makers,^2^ this can be frustrating but also empowering: experts can do something, instead of idly hoping for the best. This is in sharp contrast to the complex models of expert and policy-maker interaction developed by political and other social scientists (see Smith^2^ for a comprehensive overview). Political science theories of the policy process generally see science as only one factor influencing policy among many, and experts themselves as only one group of actors. Their advice may be eclipsed by powerful vested interests, public opinion and ideologies or competing arguments and actors.

Public policy theories generally do not consider experts as a homogeneous group separate from business and government. Rather, they are integrated within less well-defined, permeable groups such as epistemic communities, but also issue networks, policy communities, policy networks or advocacy coalitions^11–15^ (also Brooks, this issue). In addition to scientists and experts, these groups include actors from within the government and civil service, the media, civil society and business. Their ideas and policy solutions compete for dominance with other groups in policy arenas characterized by complexity, crises and external shocks. Scientific evidence, though often important, is not guaranteed to be the decisive factor. In fact, it may not even be used in rational debates, but may instead serve more symbolic functions.^16^

Among these approaches, the concept of epistemic communities stands out for the public health community for two reasons. First, it gives special importance to science and evidence as a foundation around which like-minded individuals unite. Second, it retains some of the ‘hero narrative’ of the linear knowledge transfer model in that it sees experts as key actors in the policy process. At the same time, it allows for complexity by introducing scope conditions and refining the causal mechanism by which expert influence is expected to act. The rest of this paper elaborates on the notion of epistemic communities in more detail, using their involvement in the rise of HTA in the EU as an example.

## Epistemic communities and their influence over policy

Originally developed in the early 1990s by international relations scholars, the concept of epistemic communities sought to explain instances where countries with markedly diverging interests and priorities had agreed on common policies: pollution control in the Mediterranean, nuclear arms control, a moratorium on whaling.^17–19^ These choices were explained by continued efforts of ‘networks of professionals with recognized expertise and competence […] and an authoritative claim to policy-relevant knowledge’.^3^ These epistemic communities come together around:

‘(1) a shared set of normative and principled beliefs, which provide a value-based rationale for the social action of community members;

(2) shared causal beliefs, which are derived from their analysis of practices leading or contributing to a central set of problems in their domain and which then serve as the basis for elucidating the multiple linkages between possible policy actions and desired outcomes;

(3) shared notions of validity – that is, intersubjective, internally defined criteria for weighing and validating knowledge in the domain of their expertise; and

(4) a common policy enterprise – that is, a set of common practices associated with a set of problems to which their professional competence is directed, presumably out of the conviction that human welfare will be enhanced as a consequence’.^3^

Since the 1980s, we can observe such epistemic communities emerging around HTA. HTA dates back to the 1970s when developed countries’ hospitals started buying computer tomography scanners—the prototype of an innovative and exciting but expensive technology, diffusing rapidly and uncontrollably in clinical practice.^20^ Experts from different disciplines were needed to try to predict the scanner’s consequences for patients’ health outcomes, clinical practice, medical ethics and the broader society as well as for hospital and public budgets. From the mid-1970s, health economists, public health specialists, statisticians and some clinicians met and exchanged views at international meetings, including several ‘consensus conferences’.^21^ They established specialized journals, such as *Health Technology Assessment (HTA)* and the *International Journal for Technology Assessment in Health Care.* International professional networks, such as the International Society of Technology Assessment in Health Care or the International Society for Pharmacoeconomics and Outcomes Research, were created.^22^ Although allowing for debate and disagreement, the result of this intellectual exchange was the emergence of an international community united around:

- shared normative and principled beliefs—that only treatments that are effective, or cost-effective, should be covered from public budgets;
- causal beliefs—that decisions based on evidence lead to better distribution of resources in health care;
- notions of validity of knowledge—that it is possible to assess the value of treatments and quantify or categorize their benefits, costs and other consequences for society;
- a common policy goal—to implement HTA as an answer to problems of growing health care budgets in the age of increasingly sophisticated medicine and rising patient demand.

These communities were originally international or national, with Sweden and the USA playing notable roles,^23^ but supranational European groups soon followed. At the EU level, collaborative networks on HTA date back to late 1980s.^24^ In individual member states, HTA communities sometimes emerged much later: e.g. around 2002 in Poland or 2011 in the Czech Republic.^4^

Once assembled, epistemic communities are expected to do three things to achieve their policy goal: first, actively disseminate their views and arguments. Second, gain access to decision-makers, either by gaining their attention, or by ‘[consolidating] bureaucratic power within national administrations and international secretariats’^3^ and shaping policy-makers’ preferences from within. Finally, convince decision-makers of their preferred policy solution.^4^ In practice, this causal mechanism followed its ideal outline for instance in Poland. There, the HTA community organized workshops and training for Ministry of Health and payer officials, and when its key members gained influence in the Ministry, it managed to convince the Minister that HTA was the response to urgent problems with compliance with the Transparency Directive (89/105/EEC) and defining a basic benefit package of health services.

## Conditions for success

However, things are not always that smooth. Some countries, e.g. the Czech Republic, have not institutionalized HTA, despite the epistemic community actively pursuing the goal.^4^ HTA at the EU level could also, for a long time, be seen as an example of an epistemic community’s unsuccessful efforts. HTA has had numerous proponents in Brussels, notably the European Commission, and an active network of HTA practitioners gathered in the European network for HTA (EUnetHTA). However, it had been absent from official EU policy, at least until the recent Commission legislative proposal for a Regulation on HTA.^25^ Despite longstanding reports of a European HTA agency in the making,^5^^,^^26^ repeated attempts at associating HTA with dedicated body had produced limited results: EUnetHTA has been a succession of precariously funded networking exercises, and a clause in the 2011 Patients’ Right Directive (2011/24/EU), establishing a ‘permanent HTA network’, led to a formalistic grouping of officials from member states’ health ministries, rather than a collaboration of HTA experts.^27^ Irrespective of the fate of the current Commission proposal, the road to EU-level HTA has not been straightforward.

The epistemic communities’ literature has several explanations for such cases. One answer would suggest that the causal mechanism broke down somewhere—perhaps the European HTA community never quite secured the buy-in of key decision-makers, e.g. important EU member states. Member states’ support is a necessary condition for a Commission’s proposal to become EU law and the Commission is unlikely to pursue a policy where unsurmountable opposition can be expected. It is possible that HTA had long been considered too sensitive. From assessing the occasional CT scanner, HTA has evolved into an integral part of many countries’ processes for setting prices and reimbursement levels for pharmaceuticals. As such, it touches on member states’ closely guarded national competence to govern their own health care systems. Signs of resistance ahead might have discouraged the Commission from actively pursuing Europeanization of HTA, despite its sympathy to the HTA community’s cause.

Another epistemic communities’ explanation for the long EU silence on HTA goes back to insights from advocacy coalition framework and other policy process theories, namely that epistemic communities are not the only players in town. It sees their success or failure as a matter of competition with other actors, including rival epistemic communities and interest groups,^28^ or wider forces, including public opinion and electoral politics.^29^ In the case of EU-level HTA, this would mean there could have been active opponents of HTA—or proponents of better ideas.

Yet another explanation is that the epistemic community did not satisfy some of the many ‘conditions for success’ the literature foresees for their efforts to work. These include the phase in the policy process (epistemic communities are more likely to be persuasive when defining or framing the original debates), the nature of the policy problem (epistemic communities are more likely to succeed when the issue is technocratic, as opposed to politicized) or the degree of internal cohesion and professionalism within the community.^30^^,^^31^ Perhaps the European HTA community was not cohesive enough, or maybe HTA, with its links to national pricing and reimbursement, is too politically sensitive to ever be Europeanized.

A final alternative answer focuses on the wider policy debate and the decision-makers, rather than the experts or their rivals. The key scope conditions under which scientists can be expected to influence decision-makers’ preferences have originally been formulated as uncertainty and complexity surrounding the issue.^32^ They can be extended to include decision-makers’ demand for expert input, which can have different reasons: solving a pressing problem, gaining legitimacy by invoking evidence, supporting particular policy positions, etc.^4^^,^^16^ In the case of European HTA, this would mean that relevant policy-makers (in the Commission, the European Parliament or in the member states) had possibly not felt the need to listen to the HTA epistemic community—there had not been any crises or urgent problems which would require advice of experts proposing HTA, or perhaps not at the EU level.

Which of these hypotheses are the most plausible to explain the long road to EU HTA is a matter for empirical research. The epistemic communities concept nevertheless provides a structure that social scientists, but also the HTA community itself, can use to guide their analysis of past events, and their expectations of future developments.

## Conclusion

Examining the evidence-policy relationship through the lens of epistemic communities, or other political science approaches to the policy process, offers a new perspective to the public health community, which often sees its efforts as part of a linear, rational knowledge transfer. In this linear model, the scientific community has significant agency and influence over the final policy formulation. As a result, it is bound to look inward to explain any failure to influence policy, and try to overcome its ‘shortcomings’ by improving its communication or simplifying its language, findings and messages. Political science tells us that communication may not be the problem: policy-makers are selective in when, why and how they ask for expertise,^33^ and they can sometimes favour alternative voices or evidence. Seeing expert input as only one part of the policy process can also explain why, even if the experts’ preferred policies are adopted, the minute details of their design and implementation do not always fully follow their advice. This can be because of intervening structural factors, such as organization of the health system or financial constraints, but also because other actors have their say, for instance competing epistemic communities or professional societies, industry and the public. Analyzing its efforts as part of a wider policy-making process could help the public health community make peace with the occasional frustration, and structure its learning from past experience.

## Funding

This open access Supplement received funding under an operating grant from the European Union’s Health Programme (2014–2020).

*Conflicts of interest*: None declared.

**Key points**

- Public health experts often see their efforts to influence policy as a linear, rational “hero’s journey” of their ideas into policy (i.e. “knowledge transfer”).
- The political science concept of epistemic communities allows an alternative perspective.
- Epistemic communities are groups of experts with a common policy goal derived from their shared knowledge.
- They operate in contexts of complexity where other actors compete to convince policy-makers of their preferred policy solutions.
- Analyzing this complexity could help the public health community to better target their future policy efforts.
